# Ethics guidelines for environmental epidemiologists: 2023 revision

**DOI:** 10.1097/EE9.0000000000000299

**Published:** 2024-03-12

**Authors:** Ruth A. Etzel, Nivine H. Abbas, Michael P. Anastario, Adetoun Mustapha, Olayinka Osuolale, Atanu Sarkar, Ireneous N. Soyiri, Emile Whaibeh, Colin L. Soskolne

**Affiliations:** a Milken Institute School of Public Health, The George Washington University, Washington, District of Columbia; b Public Health Department, Faculty of Health Sciences, University of Balamand, Balamand, Lebanon; c Department of Health Sciences, Northern Arizona University, Flagstaff, Arizona; d Nigerian Institute of Medical Research, Lagos, Nigeria; e Department of Biological Sciences, Elizade University, Ilara Mokin, Nigeria; f Division of Community Health and Humanities, Faculty of Medicine, Memorial University of Newfoundland and Labrador, St. John’s, Newfoundland and Labrador, Canada; g Faculty of Health Sciences, Hull York Medical School, Institute for Clinical and Applied Health Research, University of Hull, Hull, United Kingdom; h Saint-Joseph University of Beirut, Beirut, Lebanon; i University of Alberta, Edmonton, Alberta, Canada

**Keywords:** Accountability, Community engagement, Conflict of interest, Institutional review board, Community-based participatory research, Professional standards, Normative standards of practice

## Abstract

Recognition of the importance to environmental epidemiology of ethical and philosophical deliberation led, in 1996, to the establishment of Ethics Guidelines for the profession. In 1999, these guidelines were adopted by the International Society for Environmental Epidemiology. The guidelines were revised in 2012 and again in 2023 to ensure continued relevance to the major issues facing the field. Comprising normative standards of professional conduct, the guidelines are structured into four subsections: (1) obligations to individuals and communities who participate in research; (2) obligations to society; (3) obligations regarding funders/sponsors and employers; and (4) obligations to colleagues. Through the 2023 revision of the Ethics Guidelines, the International Society for Environmental Epidemiology seeks to ensure the highest possible standards of transparency and accountability for the ethical conduct of environmental epidemiologists engaged in research and public health practice.

**What this study adds:**

This update to the Ethics Guidelines seeks to ensure the highest possible standards of transparency and accountability for the ethical conduct of environmental epidemiologists.

Environmental epidemiology faces significant ethical challenges because of the involvement of powerful stakeholders whose influence may affect all levels of research and policy formulation. Although findings of environmental epidemiology studies play a critical role in underpinning evidenced-based policies aimed at protecting the public from environmentally determined harms, epidemiologic findings also can have direct effects on industry profits, careers, academic funding, and professional advancement. Conflicting interests have become rampant, and ethically challenging situations can threaten the core tenets of the discipline, which focuses on maintaining, enhancing, and promoting health in communities worldwide.

Environmental epidemiology functions within a social and political context in which laws, technology, economic pressures, and social norms are evolving. Thus, it behooves the field to maintain its guidelines for ethical practice. Guidelines are an essential instrument in any professional discipline, not only to serve as a guide to normative practices but also as a standard against which professionals could be held to account. The International Society for Environmental Epidemiology first adopted Ethics Guidelines in the late 1990s, based on work by Soskolne and Light.^1^ The Guidelines are maintained by its Ethics and Philosophy Committee, one of the earliest, active, and enduring ethics committees in the field of epidemiology. Since its inception in 1991, the committee has taken an active role in supporting ethical conduct and promulgating Ethics Guidelines for the profession.

The Ethics Guidelines for Environmental Epidemiologists address the four major categories of ethical conduct: obligations to individuals and communities who participate in research, obligations to society, obligations regarding funders/sponsors and employers, and obligations to colleagues. The first revision to the guidelines was produced and adopted in 2012.^2^ This commentary serves to bring to the attention of environmental epidemiologists the fact that the International Society for Environmental Epidemiology adopted the second revision of its Ethics Guidelines in September 2023. They are accessible at https://www.iseepi.org/docs/ISEE_Ethics_Guidelines_Adopted_17_Sept_2023.pdf.

This major update of the guidelines was prompted by certain trends and growing research challenges, including a sharp increase in reports of conflicts of interest (>1.7 million references in a 2023 PubMed search); an increase in retractions of scientific articles (>7,000 retractions found in a 2023 PubMed search); an increase in industry-funded research at academic institutions (and a concomitant increase in the proportion of academic faculty supported by or funded by industry); destruction of the natural environment and the climate crisis; industry and economic stakeholder influence on government policies; big government interference in research and the dissemination of findings that hamper the independence of researchers; environmental injustice; imbalances in the allocation of research funding and priorities; questions about data access and ownership of public health information; unacceptable behavior among some scientists and whistleblowing by other scientists; the globalization of public health issues that require collaborative professional efforts to address them; and the increasing use of artificial intelligence. It is noteworthy that some of the major trends and research challenges identified at the time of the 2012 revision remain challenges today. The subcommittee that developed the 2023 update of the guidelines focused on including current and evolving ethical and philosophical challenges, and recrafting recommendations. A core writing group (R.A.E., N.H.A., M.P.A., A.M., O.O., A.S., I.N.S., E.W., and C.L.S.) took primary responsibility for conducting the review and developing revisions, with input invited from many other members of the Ethics and Philosophy Committee. Over a 1-year period, and through an iterative process, suggested revisions and updates were evaluated and incorporated by consensus of the core writing group and the Ethics and Philosophy Committee. The entire process was informed by a workshop “Ethical perspectives on accessing and sharing environment and health data” organized on 11–12 November 2022.

Once the draft guidelines were accepted by a majority vote of the Ethics and Philosophy Committee, they were submitted to the Executive Council of the International Society for Environmental Epidemiology for comment and review. The final version was accepted and adopted by the Executive Council in September 2023. The current revision has been translated into Spanish and is accessible at: https://www.iseepi.org/docs/Espanol_ISEE_Ethics_Guidelines_Adopted_16_Sept_2023.pdf.

In the future, the committee plans to translate the guidelines into Arabic and French.

## Key components of the guidelines

The aim of this commentary is to outline the components of the guidelines and to highlight some major changes since 2012. One major change is that, for the first time, the guidelines emphasize the intrinsic value of the natural environment, (including nature, ecosystems, and biodiversity). Second, the guidelines highlight the ethical responsibility of environmental epidemiologists not only to engage in objective scientific inquiry, but also to recommend measures to prevent negative health outcomes and to promote measures to protect both the environment and public health locally, regionally, nationally, and globally. Third, the guidelines point out that, along with the environment and all that it sustains, environmental epidemiologists value human life and human dignity. Fourth, the guidelines address the ethical concerns with using publicly available datasets and technologies.

The guidelines are structured into four major sections with key subsections. The structure of the four topics has not changed from the original guidelines because these four areas of ethical consideration remain foundational to environmental epidemiology research and practice. The four obligations are to individuals and communities, to society, to funders/sponsors and employers, and to colleagues.

## Obligations to individuals and communities who participate in research

Epidemiologists and supporting institutions are obliged to recognize the rights of research participants. This expectation is not unique to environmental epidemiology; it reflects standard bioethical principles.

Four primary themes delineate these obligations:

1. Research should avoid harm to the individuals and communities who participate. Knowledge gained should be disseminated widely, and the benefits gleaned should be accessible to the communities who participate. This topic covers the concepts of (i) beneficence (i.e., doing good), (ii) the precautionary principle, (iii) nonmaleficence (i.e., doing no harm), (iv) respect for autonomy (i.e., the individual’s right to self-determination), (v) community input in the research process, (vi) full disclosure of risks and benefits, and (vii) prompt disclosure of results.
2. Informed consent before research is initiated. This core ethical consideration addresses (i) individual rights, (ii) public communication, (iii) consent for biospecimens, (iv) cultural sensitivity of consent, (v) financial disclosure of all sources of financial support, (vi) financial conflict verification, (vii) confidentiality of public data and records, (viii) data available on the internet, and (ix) types of informed consent.
3. Confidentiality. A framework for assuring confidentiality includes the need for a confidentiality plan and data security, avoiding identification of individual participants, sharing of confidential information, and extraordinary circumstances where breach of confidentiality may be justified.
4. Review of research protocols by institutional review boards (IRBs) or equivalent oversight committees. The critical role of IRBs, or their equivalent, in the review and oversight of research, is discussed, including (i) IRB roles and responsibilities, (ii) local values in ethics oversight, (iii) ethics and study design, (iv) principal investigator’s responsibility for ethical practice, and (v) conflicting interests of IRB reviewers.

## Obligations to society

The guidelines emphasize epidemiologists’ obligations to society, addressing several important ethical considerations that may affect this fundamental responsibility:

1. Avoiding partiality. Whether conscious or unconscious, partiality should be avoided, impacting the choice of research methods and communication of results, inappropriate interference in research, and avoidance of bias.
2. Avoiding conflicting interests. There is a growing threat to research integrity fueled by conflicting interests. This section emphasizes the need to avoid conflicting interests, provide full disclosure of financial or other relationships, and ensure transparency in disclosures.
3. Conduct that facilitates just environmental health policy and practice. We acknowledge the need for (i) recognition of different ethical worldviews, (ii) causal inference, (iii) contextualization of research results, (iv) guidelines for reanalysis of data, (v) advocacy, (vi) distributive justice, (vii) research priorities as a reflection of public health burden, (viii) data sharing in the public interest, (ix) data protection in the public interest, (x) respect for the natural environment, (xi) long term impacts, (xii) choice of methods and practice, and (xiii) outcome measures.
4. Community involvement. The key aspects of community involvement focus on engagement of stakeholders, partnerships, and conveying information of uncertain biological significance.
5. Communication and action plan. This aspect of research practice includes (i) reporting of research findings, (ii) communication with the media, (iii) transparency regarding assumptions and uncertainties, (iv) communications and action plan, (v) avoidance of misrepresentations and improper inferences, and (vi) mental health impact of research results.

## Obligations regarding funders/sponsors and employers

There is sometimes a tension between the interests of various stakeholders and the primary public health goals of environmental epidemiologic research. The guidelines address core principles that may serve as a guide in these circumstances.

1. Specifying obligations. To protect research integrity, we should evaluate the motivations of stakeholders to protect the public interest, communicate obligations to funders and employers, and avoid funding or other undue influence on research methods or results.
2. Protecting privileged information (including intellectual property and trade secrets). Privileged information may be used in the conduct of research, provided that permission is granted and confidentiality restrictions are maintained.

## Obligations to colleagues

As members of a diverse research community, environmental epidemiologists should maintain respect and fairness toward colleagues. Often, these issues are the most difficult to confront because they may affect personal and professional relationships. The guidelines highlight key considerations, including

1. Specifying obligations. The guidelines address the importance of respect for intellectual property and research ideas, fair attribution, avoidance of conflicting interests, and misappropriation of research ideas. There is a need to translate knowledge gained, provide support and mentoring, respect diversity and integrity, and promote professional development while respecting new or controversial ideas.
2. Reporting methods and results. Reporting should enable assessment and replication of results, allow independence and neutrality, be subjected to peer review, and support the objectivity of reviewers.
3. Confronting unacceptable behavior. Appropriate means of confronting improper practices among colleagues are supported, including the role of international review panels to review alleged misconduct, and protection of whistleblowers. Environmental epidemiologists need to speak up against unacceptable behavior, report incidents to management, provide support to colleagues, promote a culture of respect and inclusion, and develop moral courage.
4. Communicating ethical requirements among colleagues and other stakeholders. Ethical requirements that are applicable to research and practice should be shared with colleagues, research staff, funders, and practitioners, with adequate funding and support.

## Incorporating ethics guidelines into training and practice

These guidelines are meant to provide a framework, rather than a set of rules or an ultimate solution, as we confront ethical tensions. We recognize that guidelines cannot be enforced; they serve rather as a reference and pathway for ethically conscious professionals in our field who are seeking to improve the integrity of their research or to resolve ethical challenges that they face in their work. Ethics are relative, and some concepts that are acceptable in Western societies, for example, might not be relevant or applicable in other cultures. Guidelines provide practical approaches that can help maintain the fundamental tenets of our discipline and provide thoughtful researchers and practitioners a point of reference for decision-making in an environment laden with complex pressures.

New generations of researchers and professionals are becoming more aware of and interested in this discipline, its impact on their work, and in working in inter-and trans-disciplinary ways; they recognize that research ethics can be realized only if the International Society for Environmental Epidemiology Ethics Guidelines are actively incorporated into training programs, included in institutional practices and standards, integrated into presentations or discussions at professional meetings, and promulgated as a constructive set of principles to protect the integrity and values of the profession.^3^

All those engaged in environmental epidemiology—researchers, academics, consultants, governmental and nongovernmental workers—are encouraged to make themselves, and their students and mentees, familiar with the contents of the revised Ethics Guidelines. Any suggestions to make changes to these Guidelines should be sent to the Corresponding author who will collate them for a future revision.

## Conflicts of interest statement

The authors declare no financial conflicts of interest. All authors are nonpaid members of the Ethics and Philosophy Committee of the International Society for Environmental Epidemiology. R.A.E. and A.M. are elected members of the Executive Council of the International Society for Environmental Epidemiology and receive no compensation as members of the Executive Council.

## References

[1] Soskolne CL Light A. Towards ethics guidelines for environmental epidemiologists. Sci Total Environ. 1996;184:3–3.

[2] Kramer S Soskolne CL Mustapha BA Al-Delaimy WK. Revised ethics guidelines for environmental epidemiologists (Editorial). Environ Health Perspect. 2012;120:3–3. Available at: http://www.ncbi.nlm.nih.gov/pmc/articles/PMC3440101/pdf/ehp.1205562.pdf. Accessed 27 February 2024. 10.1289/ehp.1205562 PMC3440101 22853932

[3] Soskolne CL Sieswerda LE. Implementing ethics in the professions: examples from environmental epidemiology. Sci Eng Ethics. 2003;9:3–3. 12774650 10.1007/s11948-003-0005-1

